# Treatment of traumatised refugees with basic body awareness therapy versus mixed physical activity as add-on treatment: Study protocol of a randomised controlled trial

**DOI:** 10.1186/s13063-015-0974-9

**Published:** 2015-10-22

**Authors:** Maja Sticker Nordbrandt, Jessica Carlsson, Laura Glahder Lindberg, Hinuga Sandahl, Erik Lykke Mortensen

**Affiliations:** Competence Centre for Transcultural Psychiatry, Mental Health Centre Ballerup, Maglevænget 2, 2750 Ballerup, Denmark; Section of Occupational and Environmental Health, Øster Farimagsgade 5, building 5, 1., 1353 København K, Denmark

**Keywords:** Refugee, PTSD, Depression, Trauma, Pain, Basic Body Awareness Therapy, Physiotherapy, Exercise

## Abstract

**Background:**

Treatment of traumatised refugees is one of the fields within psychiatry, which has received little scientific attention. Evidence based treatment and knowledge on the efficiency of the treatment for this complex patient group is therefore scarce. This leads to uncertainty as to which treatment should be offered and potentially lowers the quality of life for the patients.

Chronic pain is very common among traumatised refugees and it is believed to maintain the mental symptoms of trauma. Hence, treating chronic pain is believed to be of high clinical value for this patient group.

In clinical studies, physical activity has shown a positive effect on psychiatric illnesses such as depression and anxiety and for patients with chronic pain. However, scientific knowledge about physical activity as part of the treatment for traumatised refugees is very limited and no guidelines exist on this topic.

**Methods/design:**

This study will include approximately 310 patients, randomised into three groups. All three groups receive psychiatric treatment as usual for the duration of 6–7 months, consisting of consultations with a medical doctor including pharmacological treatment and manual-based Cognitive Behavioural Therapy. The first group only receives treatment as usual while the second and the third groups receive either Basic-Body Awareness Therapy or mixed physical activity as add-on treatments. Each physical activity is provided for an individual 1-hour consultation per week, for the duration of 20 weeks. The study is being conducted at the Competence Centre for Transcultural Psychiatry, Mental Health Centre Ballerup in the Capital Region of Denmark.

The primary endpoint of the study is symptoms of Post Traumatic Stress Disorder; the secondary endpoints are depression and anxiety as well as quality of life, functional capacity, coping with pain, body awareness and physical fitness.

**Discussion:**

This study will examine the effect of physical activity for traumatised refugees. This has not yet been done in a randomised controlled setting on such a large scale before. Hereby the study will contribute to important knowledge that is expected to be used in future clinical guidelines and reference programs.

**Trial registration:**

ClinicalTrials.gov NCT01955538. Date of registration: 18 September 2013.

**Electronic supplementary material:**

The online version of this article (doi:10.1186/s13063-015-0974-9) contains supplementary material, which is available to authorized users.

## Background

The treatment of traumatised refugees comprises one of the fields of psychiatry with the least scientific knowledge [1, 2]. Thus, there is a serious lack of systematic research on treatment outcomes in traumatised refugee populations, and the lack of evidence motivated the present study.

By far the majority of studies of posttraumatic stress disorder (PTSD) have focused on road victims, rape victims and war veterans. There is reason to believe that the possibility of benefiting from treatment is related to the range and nature of the trauma that underlies the trauma-related disorder. Consequently, it is a problem that the four existing Cochrane analyses on treatment effect on PTSD among adults only include very few studies based on refugee populations [1–4].

Evidence based treatment for this complex patient group is therefore scarce. This leads to uncertainty concerning the treatment which should be offered and potentially lowers the quality of life of the patients [5].

The overall research question of this study is to evaluate if adding physical activity to standard psychiatric treatment improves the mental health, quality of life and functioning of traumatised refugees. Thereby, the focus of this study is on the relationship between physical activity and PTSD, including effects of physical activity on the comorbidities of PTSD, one of which is chronic pain.

Chronic pain is a commonly seen co-morbidity to PTSD [6–10]. According to the well-established "Mutual Maintenance Theory", PTSD and chronic pain will mutually maintain the symptoms of one another [8]. Consequently, treatment of chronic pain plays a central role in improving the treatment and wellbeing of patients with PTSD. Nevertheless, little scientific knowledge exists on how to treat chronic pain in traumatised refugees.

Biological and psychological theories have hypothesised on and clinical studies have examined the effects of physical activity on other psychiatric illnesses than PTSD [11–18], including some of the important co-morbidities to PTSD. Regarding depression, a central comorbidity to PTSD [19–24], the evidence is based on few methodically robust studies and systematic reviews which suggest small or moderate effects of physical activity [21, 25, 26]. Importantly, positive effects of physical activity have also been seen for chronic pain [27–35].

However, scientific knowledge about physical activity as part of the treatment for traumatised refugees is very limited and no national or international guidelines exist on this topic [2, 5, 36]. Despite this, physical activity in various forms is often used as part of the treatment for traumatised refugees. Just as it is the case for depression, the potential working mechanisms behind the effect of physical activity on symptoms of PTSD are unclear.

Only a few randomised controlled trials on traumatised refugees with PTSD investigate physical activity as the intervention. A small randomised controlled trial published in 2011 on the influence of physical activity on chronic pain and symptoms of PTSD based on the "Mutual Maintenance Theory" [37], examined the effects of mixed physical activities (exercises of stretching, strength and endurance) as additive therapy to psychoeducation and biofeedback-based cognitive behavioural therapy. The study showed larger effect sizes for coping with pain and for improvements in symptoms of anxiety in the physically active group, suggesting specifically that physical activity is a valuable addition to pain management for traumatised refugees [37]. However, the study is small (n = 30) and these preliminary results need replication in a larger trial [37].

Basic Body Awareness Therapy (BBAT) is one type of physical activity, which has been studied as intervention in a number of trials on other illnesses such as chronic pain, fibromyalgia, schizophrenia, personality disorders and non-specific musculoskeletal disorders [14, 16, 29–31, 38]. BBAT is a modification of BAT (Body Awareness Therapy) and was first described by the French psychoanalyst and actor, J. Dropsy [39]. It focuses on the basic function of movements related to posture, coordination, free breathing and awareness [29]. The aim of Basic BAT is to integrate the body in the total experience of the self and to restore body awareness and body control [40]. As for the type of physical activity, BBAT is gentle and non-strenuous; which is another reason why it could be suitable for traumatised refugees.

In order to test the feasibility of BBAT on a group of traumatised refugees, a pilot study was conducted in 2012 [41] at the Competence Centre for Transcultural Psychiatry (CTP). In this pilot study, Arabic-speaking patients were treated with weekly BBAT sessions in groups, for a period of 13 weeks. Fourteen patients were included and 9 patients (5 men and 4 women) completed the BBAT treatment. The study was primarily qualitative and semi-structured interviews were conducted with all patients. The participants showed high acceptability, compliance and satisfaction with BBAT, and this was also the case in a previous study on patients with prolonged musculoskeletal disorders [42]. Some patients in the CTP pilot study chose not to participate due to the group element.

The Cochrane review; ”Sports & Games for post-traumatic stress disorder” from 2010 concludes: “Randomised controlled trials assessing the effect of sport and game interventions are needed to inform the current practice of using sports and games to improve symptoms of PTSD” [2]. This is in line with the results of more recent above-mentioned studies and points to the fact that there is a need for more systematic research examining a number of unsolved issues: 1) how effective is physical activity as a treatment of symptoms of PTSD and of chronic pain; 2) what are the relative benefits of different types of physical activities; 3) whether group or individual physical activity is the most efficient; 4) what is the optimum duration of physical activity; 5) what intensity does the physical activity need to have in order to have an effect.

### Objectives

The objectives of this study are 1) to examine the differences in treatment outcome of patients treated respectively with or without physical activity as an add-on treatment to psychiatric treatment as usual (TAU); 2) to study if BBAT has a higher impact on the outcome measures compared to mixed physical activity; 3) to investigate if an increase in physiological parameters such as strength, endurance, balance and coordination correlates with an improvement in mental health; 4) to examine if the number of hours spent on home exercises with the planned physical activity is a positive predictor of the treatment effect.

## Methods

This study is a parallel group superiority study with an allocation ratio of 1:1:1. The study is a randomised controlled trial and will include approximately 310 traumatised refugees in the period from September 2013 to September 2015. Of those patients a minimum of 200 are expected to complete the trial in accordance with the protocol.

The trial setting is the Competence Centre for Transcultural Psychiatry (CTP), at Mental Health Centre Ballerup, situated in the Capital Region of Denmark. All doctors, psychologists and physiotherapist working on the project are working according to manuals, and have been trained in the use of the manuals. The manuals have been developed specifically to the patient group of traumatised refugees. Regarding the physical activity, only physiotherapist certified with BBAT competence are treating patients with BBAT. For further and more detailed description of CTP and its working methods, see the recently published articles [43, 44].

Concerning adherence to the protocol (regardless if it is a consultation with a doctor, psychologist or physiotherapist), the methods or themes and exercises used in each session are registered in the medical record of the patient immediately after the treatment session. Regular meetings with the physiotherapists are being held to monitor and ensure quality of the research-related treatment and data collection and discuss manual related issues.

The patients are being randomised into three groups: all three groups receive TAU (see the later section about TAU). While one of the three groups is a control group and solely receives TAU, the two other groups (both active comparator groups) receive add-on treatment in the form of physical activity. One of the groups is assigned to Basic Body Awareness Therapy (BBAT) while the other group is assigned to mixed physical activity (MPA) (see the later section about the specific interventions). For all three groups the treatment period is approximately 6–7 months.

In the study only the outcome assessors carrying out the Hamilton Depression and Hamilton Anxiety rating (Hamilton D + A) [45] are blinded. These outcome assessors only know that the patients are in the study but are not aware of the allocated treatment. Unblinding is not necessary. In a potential emergency situation such as severe suicidal thoughts during depression rating the responsible doctor or another medical doctor will be called for without need for unblinding the Hamilton rater.

The outcome assessors carrying out the observer rated Hamilton D + A are trained medical students and they participate in regular training sessions to ensure high interrater variability.

**Fig. 1:**
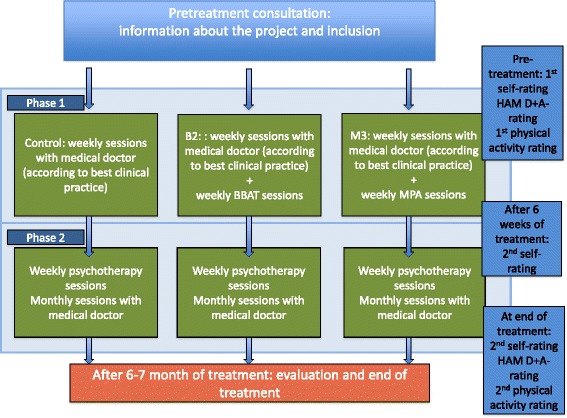
Course of treatment and data collection. The course of treatment consists of phase 1 and phase 2. Both self-administered ratings and observer-ratings (blinded as well as well as non-blinded) will be applied in the study

The assessment of the majority of outcomes takes place three times during the treatment period where the patients fill out self-administered rating scales: once at the pre-treatment consultation (baseline), once halfway through the treatment (before the first psychotherapy session) and once just after finalising the treatment (see also Additional file 1 and Fig. 1). Furthermore the medical doctor also fill out a rating scale (Global Assessment of Functioning and Global Assessment of Symptoms [46]) three times during the course of treatment as well as HoNOS before and after the course of treatment [47]. The ratings and tests specifically related to the physical activity will take place independently from the other ratings at the beginning and at the end of the assigned intervention of physical activity. All ratings scales and tests (physiotherapeutic as well as psychological) will be performed and filled out by the patients in all three treatment groups, regardless whether the patient is in one of the active comparison groups or in the control group. The blinded observer-ratings of depression and anxiety are carried out at the start and at the end of the treatment period. The applied rating scales are described in the outcome section below.

The primary endpoint of the study will be symptoms of PTSD, and secondary endpoints will be depression, anxiety, and quality of life, functional capacity, coping with pain, body awareness and physical fitness. For an illustration of the course of the study, see Additional file 1 and Fig. 1.

If needed, an interpreter will be present at each consultation during all parts of the intervention. The patient will have one assigned interpreter that follows the patient during the entire course of treatment. Interpreters are being used for approximately 70 % of the consultations. All interpreters are used to working within the setting of CTP where research and treatment are integrated and they know the questionnaires and rating scales well. An introduction meeting is held with the interpreters at the start of the trial to ensure their understanding of the research project and the methods used in the project.

### Participants

The study aims at including 310 patients of which 200 are expected to complete the treatment (based on experiences from the previous randomised trials carried out at CTP). The inclusion period is planned to last for approximately 2 years.

The patients included in the study are all adult (≥18 of age) traumatised refugees or traumatised persons who have been family renunified with a refugee. Regarding the background/nationality of the patients, we expect a representation which is relatively similar to what has been shown in a recent study of the patients at CTP [48]. This implies that the majority of patients are expected to originate from the Middle East, with the biggest proportion originating from Iraq followed by Palestinians, Afghans and Iranians. Patients from Bosnia and Serbia are also expected to be well-represented [48]. Patients are referred to the clinic either by their general practitioner or by a referring doctor in a specialist unit. If the patient is considered likely to be in the target group for treatment at CTP, the patient is invited for a pre-treatment assessment with a medical doctor. The experience from the former randomised clinical trials conducted at CTP tells that a high percentage of the patients are positive towards participating in research projects and no special strategies are used to achieve the adequate number of participants.

The participants are not receiving any provision or compensation for participation in the study. However all participants are subjected to “The rights of a trial subject in a biomedical research project” by the Danish National Committee on Biomedical Research Ethics [49].

#### Inclusion criteria

The inclusion criteria for the trial are the following: Patients must be referred to treatment for PTSD at CTP from September 2013 to September 2015; age 18 or older; must be refugees or persons who have been family reunified with a refugee; have PTSD according to the ICD-10 research criteria; psychological trauma in the past; assessed by a medical doctor to be motivated for treatment; provide written informed consent.

#### Exclusion criteria

Patients will be excluded from the trial if they have a severe psychotic disorder (defined as patients with an ICD-10 diagnosis F2x and F30.1-F30.9); are current abusers of drugs or alcohol (F1x.24-F1x.26); are physically disabled in a way which prohibits participation in the physical activities; have cardiac arrhythmia identified on the electrocardiogram taken before start of the treatment or symptoms of heart problems that are unclarified.

### Treatment groups

All three groups receive psychiatric treatment as usual (TAU). The control group is assigned to TAU alone (see the later section about TAU). The two other groups are assigned to a combination of TAU and add-on treatment consisting of either Basic Body Awareness Therapy or mixed physical activity respectively. The add-on treatment starts approximately 2 weeks after the beginning of TAU and is provided individually.

The course of treatment and procedure for the ratings are illustrated in Additional file 1 and Fig. 1. A detailed description of the intervention is given below.

### The intervention and the course of treatment in an overview

The intervention consists of physiotherapeutic treatment, in which the two active comparator groups are being assigned to each type of physiotherapeutic treatment as add-on treatment to the treatment as usual (TAU).

### Treatment as usual (TAU)

TAU overall consists of 6–7 months of medical treatment according to best clinical practice in the field and manual-based CBT.

TAU is divided into two phases and consists of the following two elements:

#### Consultations with a medical doctor

As part of TAU each patient is offered 10 consultations with a medical doctor. During phase 1 (the first two months), these consultations take place approximately once a week and are primarily concerning medication and psychoeducation. If indicated, the medical doctor will as part of TAU initiate and monitor pharmacological treatment, and the psychoeducation includes a wide range of topics such as symptoms of PTSD and depression; the rationale of treatment; physical activity, bodily reactions and anxiety; how PTSD affects the brain; sleep disturbances; healthy life style, including a proper diet and exercise; breathing and relaxation exercises, and chronic pain. One-page hand-outs are available on all the topics in five different languages. The hand-outs are given to the patient after having been discussed during a consultation.

The psychoeducational treatment is thoroughly described in the treatment manual followed by all doctors.

During the consultations, medication is initiated in accordance with the medical treatment algorithm of the clinic, which constitutes the pharmaceutical part of TAU. The algorithm is based on the present knowledge on treatment of traumatised refugees [5, 50, 51]. In accordance with this, sertraline is first choice medication, and venlafaxine second choice of medication. Furthermore mianserine in small doses can be added if the patient has sleep disturbances as a central problem.

Phase one is followed by phase two (the next four months) where the consultation with the medical doctor takes place once a month.

In case extra consultations are needed (e.g. when important psychosocial or medical problems arise) these are noted in the patients´ medical record.

#### Psychotherapy by a psychologist

A psychologist carries out 16 sessions of approximately 1 hour in a treatment period of four months according to the CBT manual. The manual has been made specifically for this patient group and the methods, which are being used, have been adapted to fit the patient group [52]. All psychologists have all been trained in using the manual.

### Physiotherapeutic intervention

Regardless of the assigned physical activity, the participants will be scheduled to one weekly, individual session for a total of 20 weeks. One session lasts approximately 1 hour. Every patient is assigned one regular physiotherapist during the whole course of treatment. To ensure consistency regarding the treatment both physiotherapeutic interventions are manualised and the physiotherapists have been trained in using the manuals. Both physiotherapeutic interventions have a method sheet outlining the different topic/themes, which the physiotherapy should cover (e.g. “strength”, “endurance”, “balance” etc. for Mixed Physical Activity, as well as “Body Scan”, “Rotation”, “The Wave” etc. for Basic Body Awareness) with a number of specific exercises connected to every topic/theme. At the weekly session the physiotherapist will register the attendance of each patient, the exercises used during the session and the patient’s individual degree of participation in each exercise. Furthermore the amount of homework reported by the patient will be registered. With regard to the intensity of training, both physiotherapeutic interventions are at their starting point mild forms of training suitable for the patient group. Meanwhile, there is a certain flexibility to adapt the intensity to the physical capacity and/or physical limitations of the individual patient.

All patients in all three intervention groups are tested with objective physiotherapeutic assessment instruments and tests as well as self-administered rating scales at the beginning and end of the course of the physiotherapeutic treatment (see the section about outcomes below).

#### Basic body awareness therapy (BBAT)

BBAT is a body-oriented physiotherapeutic approach. BBAT uses a perspective on physiotherapy treatment directed towards an awareness of how the body is used, in terms of body function, behaviour and interaction with the self and others [53]. For more description of BBAT, see also the description of BBAT in the section “Background” above.

Only physiotherapist certified with BBAT competence will carry out the teaching of BBAT.

#### Mixed physical activity (MPA)

Physiotherapists with experience in working with traumatised refugees assisted developing the manual for the teaching in MPA. The activities include exercises covering themes of strength, endurance, balance and coordination. As described in the section above, the specific exercises are described in the manual and the actual exercises and level of participation performed in a session will be noted in the record of the patient.

#### Home exercises

Regardless of the assigned physical activity the physiotherapist will motivate the patients to do home exercises. The home exercises will be specific exercises from the type of physical activity that the patient is assigned to and will be demonstrated in the training sessions. After every session, the physiotherapist records the reported homework in the patient medical chart.

#### Concomitant care

The participants will not be referred from CTP to other physical activities during their participation in the trial. If they at the beginning of the study are already in some sort of physiotherapy/physical activity this is not discontinued during the trial.

#### Discontinuation of the intervention

Discontinuation of the allocated intervention will take place if the patient wishes to stop participation in the physical exercise, if the patient cannot at all participate in the given physical exercise because of e.g. severe pain or if a physical illness is diagnosed during the trial where physical exercise is contraindicated.

### Outcomes

Both self-administered ratings and observer-ratings (blinded as well as well as non-blinded) will be applied in the study. For details about the specific time points of the different outcomes, see Additional file 1.

The outcomes have been chosen based on perceived clinical relevance and from the perspective of enabling comparison of the results of the study with results from other studies made on the same patient group. A number of studies on traumatised refugees have applied many of the same outcomes as the present study, such as HTQ, ([37, 44, 57, 54–58], GAF [44, 52, 56, 59], HSCL-25 [44, 56, 60], HAM D + A [44, 56, 61], Symptom Checklist-90 (SCL-90) [44, 48, 56], HSCL-25 [37, 44, 48, 60, 62–64], WHO-5 [44, 56, 61], SDS [44, 54], VAS [7, 44, 56, 61, 65], and HoNOS [66]. The selected outcomes are considered clinically relevant as they have been used in other trials on the same patient group or patients who are similar in important aspects, e.g. pain-measures used for patients with chronic non-malignant pain.

All outcome measures are scored on quantitative scales, and interventions effects and group differences can consequently be analysed as differences in means in linear statistical models. Depending on the results it may be relevant to analyse proportions with significant improvement using logistic regression..

The rating scales are all translated and available in five different languages (Danish, Arabic, English, Bosnian/Serbo-Croatian and Farsi), covering the mother tongue of more than 80 % of our patients. If a version on the mother tongue of a patient is unavailable, a translator provides a verbal translation from the language that the translator feels most familiar with.

Regarding harm outcomes, all serious adverse events happening during the trial, whether caused by the intervention or not, will consistently be registered and reported to The National Committee on Health Research Ethics.

### Primary outcome measure

The primary outcome of the study is PTSD severity as measured by by *The Harvard Trauma Questionnaire (HTQ)* [57]**.** HTQ is a self-administered rating scale assessing the severity of PTSD symptoms. It is internationally applied [67] and thoroughly validated in several different languages and adapted for refugee populations [58–60, 68, 69]. The first 16 questions of the HTQ, Part IV (symptoms part) are used to monitor PTSD symptoms. These 16 questions cover all PTSD criteria in accordance with ICD-10 as well as DSM-IV.

### Secondary outcome measures

*Hopkins Symptom Check List (HSCL-25)* is a self-administered rating scale assessing the severity of anxiety and depression symptoms [70]. It is internationally applied and thoroughly validated [60, 70–74]. This is a short version of the *Symptom Checklist-90 (SCL-90)* and consists of 25 questions, 10 regarding anxiety and 15 regarding depression. In addition to this, the somatisation part of SCL-90 is used.

The *WHO-5* is a self-administered questionnaire evaluating quality of life, and it consists of five questions [75]. The questionnaire has been used to assess the quality of life in a number of psychiatric diagnostic groups [75–79].

*Sheehan Disability Scale (SDS)* is a self-administered rating scale measuring functional impairment with regard to family, work and social network using three visual analogue scales [80]. Evaluation of the scale has shown that it is sensitive to treatment effects in psychiatric patients [81, 82].

*Global Assessment of Functioning* for symptoms *(GAF-S)* and for functioning *(GAF-F)* are numeric observer rating scales used to assess the degree of social and physical functioning in adults [46]. The scales are widely used in the field of psychiatry. They have been validated in many languages and are used frequently in clinical studies in the field [48, 83, 84]. They have been applied in studies on PTSD in a number of patient groups [85, 86] also on populations of traumatised refugees [44, 59].

*Hamilton depression and anxiety scales (HAM-D and HAM-A)* are observer rating scales assessing depression and anxiety on the basis of semi-structured interviews [45]. The HAM-D and HAM-A ratings have been used for psychiatric research [23, 82, 87] and assessment of symptoms in torture survivors [56, 63]. They do not require translation as they are based on interviews carried out by the clinician.

*HoNOS* is an observer rating scale used to measure the health and social functioning of people with severe mental illness [88], and has also been used in a clinical study on a population of traumatised refugees [47].

*Pain on Visual Analogue Scale (VAS)* is a self-administered rating scale, assessing pain intensity in the head, back, upper extremities and lower extremities with visual analogue scales [89].

*Brief Pain Inventory (BPI)* short form is a self-report instrument assessing different aspects of pain [90]. It is psychometric and linguistically validated on more than 20 languages [91].

*Multidimensional Assessment of Interoceptive Awareness (MAIA)* is a self-report instrument, assessing interoceptive body awareness [70].

Three objective physiotherapeutic instruments assessing function are being used: *Dynamic Gait Index (DGI)* to evaluate the dynamic/functional balance [92]; *Senior Fitness Test (SFT)* assessing body strength and aerobic fitness [93]; and *De Morton Mobility Index (DEMMI)*, assessing the mobility of patients with many physical limitations [94].

### Randomisation and data collection

#### Randomisation

All patients will be enrolled and randomised by the medical doctor after a two-hour referral interview in accordance with inclusion and exclusion criteria, including giving an informed, written consent to the medical doctor. At the referral interview and before randomisation, the medical doctor will determine and place the patient in the right stratification group (by gender and level of severity of PTSD symptoms: score < or > 3.2 on the HTQ) and later assign the patient to the intervention.

The randomisation sequence was computer generated by the Department of Biostatistics at the University of Copenhagen. This department is not otherwise involved in the research project. Allocation is concealed by using sealed, sequentially numbered envelopes. Two secretaries not linked to the daily work at CTP manage the envelopes and they are also physically separated from CTP. When a patient has been included in the study, the medical doctor calls the secretaries administering the randomisation envelopes, and medical doctor receives immediate information on the allocated group, which is noted in the record of the patient.

### Data collection, management, and analysis

#### Sample size and power calculations

The inclusion for the trial will be terminated on September 30 2015. This is when a total of approximately 310 patients have been invited in the trial. On the basis of the completion rate in previous similar randomised trials at CTP [10], the estimated drop-out rate is about 25–30 %. At the beginning of the trial the plan was to invite 250 patients, but due to a higher (30–35 %) drop-out rate than expected (25–30 %), it was decided to invite approximately 310 patients. A conservative estimation of expected completers is set to 200.

If 200 patients are divided into three groups of about 65 patients, power to detect a group difference corresponding to ½ SD will be 81 %, while power to detect a difference of 1 SD will be close to 100 %. Differences in quantitative outcomes less than ½ SD between the two treatments are considered to be less relevant from a clinical point of view.

Cut-off scores are available for several rating scales, and these can be used to define categorical outcome variables. If for example the proportions below cut-off for clinical case status are 50 % and 25 % in two groups, power will be close to 80 % to detect a significant group differences.

### Data collection methods

Data will be collected from self-administered questionnaires as well as observer rated rating scales. All assessors have been trained in using the relevant rating scales. All collected data will be entered via double data entry.

For patients who discontinue or deviate from intervention protocols the following outcome data will be available at baseline: sex, age, information on, in- and exclusion criteria and outcomes from the following ratings: HTQ-score, HSCL-25, GAF-F and GAF-S, SDS, WHO-5, VAS.

### Statistical analysis

Statistical analyses will be conducted in Stata 14. It is expected that pre-treatment scores will be available for more patients than post-treatment scores. To conduct intention-to-treat analyses with all patients, Full Information Maximum Likelihood (FIML) will be used in analyses, which include the post-treatment scores as outcome and the pre-treatment scores as covariate. Stata’s structural equation modeling procedure “sem” will be used to conduct these analyses which incorporate all available information including pre-treatment scores for patients without post-treatment scores. The primary and secondary quantitative outcome variables will be analysed in several models: 1) Linear regression analyses of pre-treatment scores including only indicator variables for the three groups; 2) Linear regression of post-treatment scores in models including only indicator variables for the three groups; and (3) FIML with procedure sem to analyze post-treatment scores in models including indicator variables for the three groups’ pre-treatment scores as predictors. For each analysis the significance of differences between the groups will be evaluated comparing the two intervention groups and each intervention group with the group receiving TAU only. Due to the randomisation the three groups are not expected to differ on pre-treatment scores but if regression analyses of the pre-treatment scores shows significant group differences, FIML analyses corresponding to step (3) above but including variables with pre-treatment group differences in the model will be used.

In addition to intention-to-treat analyses completer analyses will be conducted according to the same strategy described above. The non-adherence group is defined as patients not following the protocol, e.g. because of few attended treatment sessions. The per protocol population is defined as those attending ≥ 10 physiotherapy treatment sessions.

Cohen’s d will be used as effect size measure derived from the baseline standard deviation. For the primary HTQ outcome the SD is expected to be about 0.5 (on a 1–4 point scale), and thus a group mean differences of 0.25 will correspond to ½ SD.

Depending on the results of the linear analyses, cut-offs can be used on symptom scores and logistic regression analyses can be carried out with over/under cut-off as binary outcome.

A fully specified statistical analysis plan will be written before unmasking.

The final report will follow the main CONSORT guideline as well as its extension to non-pharmaceutical interventions.

The non-adherence group is defined as patients not following the protocol, e.g. because of a bigger number of non-attended treatment sessions, admission to a psychiatric hospital for longer periods/(more than a few nights) during their participation in the trial. The per protocol population is defined as those attending ≥ 10 physiotherapy sessions.

The multiplicity coming from two active arms and a control group will be addressed by making a specific comparison between the groups (as described in the statistical analysis plan).

Potential attrition bias will be sought prevented by only including patients motivated for treatment. Potential attrition bias will be treated by making drop-out analysis and intention-to-treat analysis.

### Monitoring

A data monitoring team independent of the funder and with no competing interests is monitoring the collected data and ensuring that data are being collected as planned according to protocol Furthermore, the unit of Good Clinical Practice (GCP) is performing an external monitoring of the trial and the data collection approximately every 2–3 months, although not mandatory for a non-pharmaceutical trial. An External Audit by GCP has also been performed on the trial, the process being independent from investigator, sponsor and funder.

No interim analysis or stopping guidelines have been made prior to the study.

The leadership of CTP is overseeing the trial. An advisory board is following the trial, giving advice when appropriate and needed.

### Ethics and dissemination

#### Ethical considerations

The Ethics Committee of the Capital Region of Denmark and the Danish Data Protection Agency have approved the trial protocol. The project recognises the Declaration of Helsinki II. Participation in the study is voluntary and requires informed, written consent. Participants will be informed about the trial verbally and in writing. Patients can stop treatment and leave the trial at any time and taking part in the trial is not a prerequisite for receiving treatment at the clinic. Randomisation is considered ethical, as current knowledge in this field is so scarce that randomised controlled effect studies in this area are needed. This implies that results of the study are expected to improve treatment for future patients and to stimulate to further research within a relatively short time.

Furthermore, the trial is expected to generate a socio-economic gain, as it seeks to improve treatment, thereby creating better results for the patients regarding mental health and quality of life as well as social functioning. This will not only help the patients but secondarily also affect their families. No serious disadvantages or serious adverse events are expected in any of the groups during the trial.

Personal information about potential and enrolled patients is kept in the respective record of the patient in a record room, locked behind two doors outside of normal work hours. Deleting the patient identification list, which combines the Civil Personal Registration (CPR) number with the patient-ID when the permission for data processing expires, will anonymise data. The procedures for how to handle patient information, both during and after the trial, have been approved by The Danish Data Protection Agency. MN, JC, EL and LL will gain access to the final dataset.

#### Protocol amendments

The methods described in this protocol reflect the current protocol (v 3.0 dated July 12 2013). A summary of protocol amendments is summarised in Additional file 2.

All protocol versions and amendments have been submitted to the Ethics Committee of the Capital Region of Denmark. Important changes in the protocol are also reflected in the information material sent to patients about the trial as well as on: www.clinicaltrials.gov.

#### Publications and dissemination

Positive as well as inconclusive or negative results will be published.

After completion of data analysis, three publications are planned regarding the following aspects of the study:

- The effect of physical activity as an add-on treatment to psychiatric treatment as usual for traumatised refugees.
- The mental health benefits of body awareness when treating traumatised refugees with physical activity.
- The mental health benefits of improvement of fitness parameters when treating traumatised refugees with PTSD.

Trial results are planned to be disseminated on national as well as international conferences and meetings for healthcare professionals as well as information meetings with public participation. Furthermore results are planned to be disseminated via public information channels such as e.g. information meetings, radio, newspapers, magazines etc.

Trial results will also be communicated to the participants via a letter sent by post.

The Vancouver rules for authorship will be followed. There will be no use of professional writers.

Public access to the protocol is not planned but individuals who are interested can address MN and gain access to the protocol.

This study protocol is written following the SPIRIT advice (see Additional file 3).

## Discussion

This study will examine the effect of physical activity for traumatised refugees specifically in relation to effects on mental health and on pain. This has not yet been done in a randomised controlled setting on such a large scale before. Hereby the study will contribute with important knowledge that is expected to be relevant for publishing in international journals and the results are expected to be used in future clinical guidelines and reference programs for the patient group. Through this we expect the study to have an impact of the quality and scope of future treatment for traumatised refugees.

## Trial status

Inclusion of patients for this trial has started September 13th 2013 and is expected to continue inclusion until September 30 2015 (see also Additional file 4 for "WHO Trial Registration Data Set" and Additional file 5 for "Patient information and consent form").

## Additional files

Additional file 1:
**(Enrolment schedule).** (PDF 59 kb) Additional file 2:
**(List of important prototocol amendments).** (PDF 35 kb) Additional file 3:
**(The SPIRIT checklist with pagenumbers).** (PDF 106 kb) Additional file 4:
**(The WHO Trial Registration Data Set).** (PDF 61 kb) Additional file 5:
**(Patient information and consent form).** (PDF 1761 kb)

## Abbreviations

ANCOVA: Analysis of covariance
BBAT: Basic Body Awareness Therapy
BPI: Brief Pain Inventory
CPR number: Civil Personal Registration
CTP: Competence Centre for Transcultural Psychiatry
DEMMI: De Morton Mobility Index
DGI: Dynamic Gait Index
FIML: Full Information Maximum Likelihood
GAF-F: Global assessment of functioning
GAF-S: Global assessment of functioning for symptoms
GCP: Good Clinical Practice
HAM-a: Hamilton anxiety scale
HAM-D: Hamilton depression scale
HSCL-25: Hopkins symptom check list
ICD-10: International classification of diseases-10
MAIA: Multidimensional Assessment of Interoceptive Awareness
MPA: Mixed physical activity
PTSD: Post-traumatic stress disorder
SFT: Senior Fitness Test
TAU: Treatment as usual
VAS: Visual analogue scale
