# Supplementary material for: Treatment of traumatised refugees with basic body awareness therapy versus mixed physical activity as add-on treatment: Study protocol of a randomised controlled trial
Source: Trials. 2015 Oct 22;16:477. doi: 10.1186/s13063-015-0974-9 (PMC4619210; doi:10.1186/s13063-015-0974-9)
Supplement: Additional file 1: — (Enrolment schedule). (PDF 59 kb) [file 13063_2015_974_MOESM1_ESM.pdf]

Table 1. Time schedule of enrolement

	STUDY PERIOD					
	Enrolment	Allocation	Post-allocation			Evaluation
TIMEPOINT	Week 0	Week 1	*Week 2*	*Week 6*	*Week 22*	*Week 24*
ENROLMENT						
Eligibility screen	X					
Informed consent	X					
No cardiac arythmia identified on ECG	X					
INTERVENTIONS						
BBAT			X-------------------------	-------	-----X	
M3			X-------------------------	-------	-----X	
Control Group/TAU			X-------------------------	-------	-----X	
ASSESSMENTS:						
HTQ, GAF-F+S, HSCL-25, SCL-90*, SDS, WHO-5, VAS*	X			X	X	
HAM-D+A	X				X	
HoNOS.	X				X	
MAIA, BPI, SFT, DEMMI			X		X	
