# Supplementary material for: Treatment of traumatised refugees with basic body awareness therapy versus mixed physical activity as add-on treatment: Study protocol of a randomised controlled trial
Source: Trials. 2015 Oct 22;16:477. doi: 10.1186/s13063-015-0974-9 (PMC4619210; doi:10.1186/s13063-015-0974-9)
Supplement: Additional file 2: — (List of important prototocol amendments). (PDF 35 kb) [file 13063_2015_974_MOESM2_ESM.pdf]

Table 2: Protocol version and amendments – Date and version identifier.

**Issue date**: 2013-July-12<br>**Authors:** MN; JC; ELM	Original protocol version.
**Issue date**: 2015-04-24<br>**Authors:** MN; JC; ELM	Amendment 06: Because of preliminary analysis showing a higher dropout rate than expected, the number of planned included patients is raised from 250 to 288. Therefore the trial is also extended until July 2016.
**Issue date**: 2015-08-11<br>**Authors:** MN; JC; ELM	Amendment 07: Cf. amendment 06, a higher number is being included. Because of fewer patients belonging to one specific stratification group (men with a HTQ score>3.2) were included in the trial, the inclusion period is extended until September 2015 and the number of maximal included patients is raised to 340. This ensures a big enough number of the mentioned stratification group being included, in order later to be able to make statistically sound analysis.
