# Supplementary material for: Treatment of traumatised refugees with basic body awareness therapy versus mixed physical activity as add-on treatment: Study protocol of a randomised controlled trial
Source: Trials. 2015 Oct 22;16:477. doi: 10.1186/s13063-015-0974-9 (PMC4619210; doi:10.1186/s13063-015-0974-9)
Supplement: Additional file 4: — (The WHO Trial Registration Data Set). (PDF 61 kb) [file 13063_2015_974_MOESM4_ESM.pdf]

Table 4: WHO Trial Registration Data Set

**Data category**	**Information**
Primary registry and trial identifying number	ClinicalTrials.gov NCT01955538
Date of registration in primary registry	18 September 2013
Secondary identifying numbers	H-3-2013-080, PTF4
Source(s) of monetary or material support	TrygFonden, Denmark.
Primary sponsor	Jessica Carlsson, Competence Centre for Transcultural Psychiatry, Mental Health Services in the Capital Region, Denmark.
Secondary sponsor(s)	
Contact for public queries	*MN*, MD, maja.nordbrandt@regionh.dk, +45 20158373
Contact for scientific queries	*MN*, MD, Competence Centre for Transcultural Psychiatry, Mental Health Services in the Capital Region, Denmark.
Public title	Treatment of traumatised refugees: the effect of Basic Body Awareness Therapy versus mixed physical activity as add-on treatment. A randomised controlled trial.
Scientific title	Treatment of traumatised refugees: the effect of Basic Body Awareness Therapy versus mixed physical activity as add-on treatment. A randomised controlled trial.
Countries of recruitment	Denmark
Health condition(s) or problem(s) studied	Post Traumatic Stress Disorders, Depression and Chronic pain
Intervention(s)	Active comparators: Treatment as usual (control group).<br>Experimental: Basic Body Awareness Therapy as add on treatment to Treatment as usual.<br>Experimental: Mixed Physical Activity as add on treatment to Treatment as usual.
Key inclusion and exclusion criteria	Inclusion critiria: Patients must be referred to treatment for PTSD at CTP from September 2013 to September 2015; age 18 or older; must be refugees or persons who have been family reunified with a refugee; have PTSD according to the ICD-10 research criteria; psychological trauma in the

	anamnesis; assessed by a doctor to be motivated for treatment; provide written informed consent.<br><br>Exclusion critiria: severe psychotic disorder (defined as patients with an ICD-10 diagnosis F2x and F30.1-F30.9); are current abusers of drugs or alcohol (F1x.24-F1x.26); have physical handicaps that unable participation in the physical activities; have cardiac arrhythmia identified on the electrocardiogram taken before start of the treatment or symptoms of heart problems that are unclarified.
Study type	Interventional<br>Allocation: randomized<br>Intervention model: parallel assignment<br>Masking: not blinded apart from one secondary outcome (HAM-A+B)<br>Primary purpose: treatment
Date of first enrolment	September 2013
Target sample size	310
Recruitment status	Recruiting
Primary outcome(s)	Change in Harvard Trauma Questionnaire (HTQ)
Key secondary outcomes	Change in Hopkins Symptom Check List (HSCL-25)<br>Change in Hamilton depression + anxiety (HAM D+A)<br>Change in World Health Organisation-5 (WHO5)<br>Change in Sheehan Disability Scale<br>Change in Visual Analogue Scale (VAS)<br>Change in Brief Pain Inventory Short Form (BPI)<br>Change in Multidimensional Assessment of Interoceptive Awareness (MAIA)<br>Change in Global assessment of function (GAF)
