# Supplementary material for: Treatment of traumatised refugees with basic body awareness therapy versus mixed physical activity as add-on treatment: Study protocol of a randomised controlled trial
Source: Trials. 2015 Oct 22;16:477. doi: 10.1186/s13063-015-0974-9 (PMC4619210; doi:10.1186/s13063-015-0974-9)
Supplement: Additional file 5: — (Patient information and consent form). (PDF 1761 kb) [file 13063_2015_974_MOESM5_ESM.pdf]

Information on participation in a scientific project

Project title:
Treatment of traumatised refugees: the effect of Basic Body Awareness Therapy versus mixed physical activity as add-on treatment.
A Randomised Controlled Trial.

We would like to ask you to participate in a scientific project, led by the trial responsible doctor Maja Nordbrandt and the staff at the Competence Centre for Transcultural Psychiatry (CTP). Before you decide whether you want to participate, you should fully understand what the project is about and the reason why we carry it out. Therefore, we kindly ask you to carefully read this information for participants.

During therapy it will always be possible to get an interpreter. During the referral interview at the CTP this information will be further elaborated, and you will get the opportunity to ask any question you may have about the project.
You are welcome to bring a family member or a friend to the referral interview. If you decide to participate in the project we will ask you to sign a statement of consent.
Remember that you have the right to take your time to consider before you decide to sign the statement of consent.
It is voluntary to participate in the project.
You can withdraw your consent at any time and without having to give a reason.

The aim of the project
We do not know much about the best way to treat mental problems of refugees who have been exposed to violent or unpleasant experiences during war, flight and imprisonment. Some studies point towards that a good effect can be obtained by antidepressant medicine combined with psychotherapy. It is also suggested that a combination with physical activity can be efficient. However, we do not know, what kind of physical activity that has the best influence on treating traumatic reactions of refugees.
In order to be able to offer the optimal treatment of traumatised refugees in the future, this project will explore the benefits of two different types of physical activity: Basic Body Awareness Therapy and mixed physical activity.
Basic Body Awareness Therapy is a kind of physical activity that focuses on breathing, obtaining a balanced body posture and a conscious presence.
Mixed physical activity is a kind of all-round exercise and training of strength and endurance.
The project will take place at the Competence Centre for Transcultural Psychiatry and will include approximately 310 patients during the next approximately 24 months.

Project design
As a participant in the project you will be offered the same treatment that you will be offered if you chose to not participate in the project: you will be offered treatment by a medical doctor, a psychologist and a physiotherapist. The treatment course by the medical doctor and the psychologist will last 6 months all in all. You will be offered 10 consultations with a medical doctor, 16 consultations with a psychologist and 20 treatment sessions with a physiotherapist.
If you participate in the project you will be offered physical activity either during the 6 months of treatment or after the 6 months.
If you are not participating in the project we can offer you physical activity after the 6 months.

If you agree to participate in the project, a draw will decide what kind of physical activity you will be doing. During the project we will continuously examine your symptoms, your quality of life, your capability of functioning, your body awareness and how you deal with potential pain, by having you fill out some questionnaires. If needed you will be assisted by an interpreter.
It is important that you subsequently follow the treatment and the medical doctor's instructions. During the project, the unit of Good Clinical Practice (GCP) and The National Committee on Health Research Ethics will have access to personal information in order to monitor the project.

The use of the project
As a project participant you will receive the treatment, we believe is the best for you. At the same time you will contribute to the development of a better treatment for other people, who have the same problems as you. Internationally, there is insufficient knowledge about treatment within this field. Therefore, the new results of this project are very important.

Adverse effects, risks, complications and disadvantages
There will be no discomfort related to participating in the project. The selected two kinds of physical activity are very gentle and adjusted to persons who are not used to doing physical activity.

We do not expect you to experience any adverse effects due to participation in the project. We only expect that you might experience a natural tiredness or soreness of the muscles after having used your muscles during physical activity. We ask you to tell us if you experience any health related problems during the project. If we find adverse effects that we have not already informed you about, you will of course be informed at once, and you can decide whether you wish to go on with the project.

Other treatment options
If you chose not to participate in the project, you will be offered a similar treatment at the centre, but you will not be offered physical activity until after the treatment has ended.
You can also seek treatment at another rehabilitation centre for traumatised refugees.

Exclusion from and leaving the project
You cannot participate in the project if you suffer from e.g. schizophrenia or mania, or if you have a not yet clarified heart disease, or if you have physical handicaps that hinder physical activity, or if you abuse medicine or are addicted to alcohol or drugs.
If you during the physical activity feel any discomfort which might be related to heart problems, we will ask you to take a break from that activity and contact the project responsible doctor, Maja Nordbrandt, to let her advice you and perhaps request a possible further examination of your health. If, contrary to expectations, a serious situation should occur which is followed by a need for hospitalisation, you can continue treatment here, but you will no longer participate in the project. Should the project, contrary to expectations, be stopped before time, you will be told at once.

Information about economic conditions
The project has been started on the initiative of the project responsible doctor, Maja Nordbrandt, in cooperation with consultant psychiatrist Jessica Carlsson and leading consultant psychiatrist Morten Ekstrøm at the Competence Centre for Transcultural Psychiatry. The project responsible doctor is employed at the Mental Health Services, Capital Region of Denmark and is a Ph.D. student.

Financial support is being applied from the following funds: Region Hovedstadens Psykiatri, Københavns Universitet, Psykiatrisk Center Ballerup, TrygFonden, Lundbeck-fonden, Nordea Fonden, Velux Fonden, Novo Nordisk Fonden, Fysioterapipraksisfonden and Oak Foundation. From Psychiatric Centre Ballerup the project is being supported with 141.000 kr. supplying 5 months' salary of a physiotherapist. The amount will be paid over the clinical budget, of which Competence Centre for Transcultural Psychiatry is a part.
If financial support will be achieved from others than the aforementioned funds, you will be informed about the amount. There is supposedly no connection between the project responsible doctor and the contributors.

No remuneration will be given to the project participants.

Access to project results
The employees at the GCP unit at Copenhagen University Hospital/the Public GCP units in Denmark, the cooperation partners of the GCP-units as well as The National Committee on Health Research Ethics can gain access to information from your medical record in order to verify the information registered in the project. The project is expected to be completed in 2016, and the results will be published in international, scientific journals.

We hope that this information has given you an understanding of what it means to participate in the project, and that you feel prepared to make the decision about your possible participation. We ask you to further read the enclosed material: "Subjects' rights in a health scientific research project."
If you want to know more about the project, feel free to contact medical doctor Maja Nordbrandt, Competence Centre for Transcultural Psychiatry, Maglevænget 2, opgang 14, 2750 Ballerup, maja.nordbrandt@regionh.dk, tlf. +45 38646180.

Kind regards,

Maja Nordbrandt
Project responsible medical doctor

Informeret samtykke til deltagelse i et sundhedsvidenskabeligt forskningsprojekt

Informed consent for participation in a health science research project

Research project title:

The effect of Basic Body Awareness Therapy (BBAT) versus mixed physical activity as add-on treatment for traumatise refugees. A Randomised Controlled Trial

---

Trial participant declaration of consent:
I have received written and oral information and I know enough about the purpose, the method, the advantages and the disadvantages of the trial to give my consent to participate in the trial.

I know that it is voluntary to participate and that I can withdraw my consent at any time without losing my present and future rights to receive treatment.

I give my consent to participate in the research project and have received a copy of the declaration of consent, as well as copy of the written information about the project.

Trial participant's Name: ______________________
Forsøgspersonens navn

Date: ______________________ **Signature:** ______________________
Dato *Underskrift*

You will be informed if significant new information regarding your health is disclosed during the research project. If you request **to not be informed** about significant new information regarding your health that is being disclosed during the research project, please tick this box: ☐

Would you like to be informed about the results of the research project and any consequences it might have for you?

Yes (tick the box) ☐ **No** (tick the box) ☐
Ja (sæt X) *Nej (sæt X)*

Declaration from the person providing information:
I declare that the trial participant has received oral and written information about the trial.

I believe that sufficient information has been provided in order to make a decision regarding participation in this trial.

Name of the person providing information: ______________________

Date: ______________________ **Signature:** ______________________
Dato *Underskrift*
